# Tris{2-[(furan-2-meth­yl)imino­meth­yl]-4-methyl­phenolato}cobalt(III)

**DOI:** 10.1107/S1600536811044588

**Published:** 2011-10-29

**Authors:** Chunyan Li

**Affiliations:** a College of Health Science, Wuhan Institute of Physical Education, Wuhan 430079, People’s Republic of China

## Abstract

In title compound, [Co(C_13_H_12_NO_2_)_3_], the Co^III^ ion is six-coordinated by three bidentate Schiff base ligands in a distorted octa­hedral environment. Adjacent complex mol­ecules are linked through C—H⋯O hydrogen bonds.

## Related literature

Schiff base ligands may act as a bidentate *N*,*O*- (Castillo *et al.*, 2003 ▶) and tridentate *N*,*O*,*O*-donor ligands (Erxleben & Schumacher, 2001 ▶) in coordination chemistry. For the anti­tumour activity of Schiff base–metal complexes, see: Liu *et al.* (1992 ▶); Ren *et al.* (2002 ▶) and for their anti-microbial activity, see: Panneerselvam *et al.* (2005 ▶). For background to vitamin B12, see: Randaccio *et al.* (2010 ▶). For related structures, see: Olejnik & Lis (1994 ▶); Ray *et al.* (2008 ▶); Sari *et al.* (1997 ▶). For standard bond lengths, see: Allen *et al.* (1987 ▶).
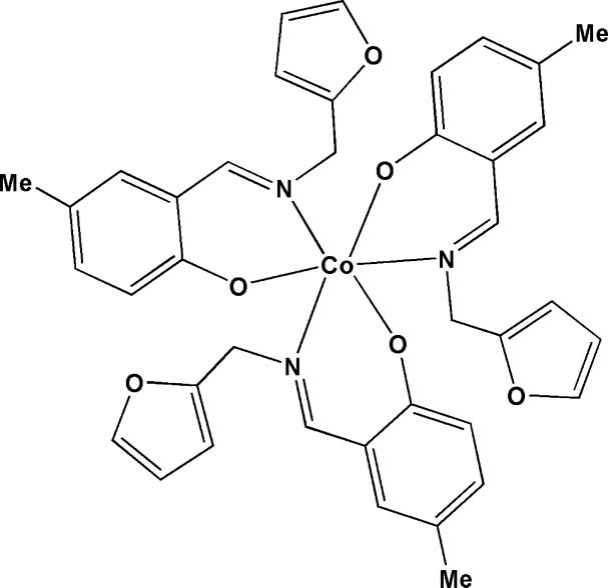


## Experimental

### 

#### Crystal data

[Co(C_13_H_12_NO_2_)_3_]
*M* *_r_* = 701.64
Triclinic, 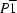

*a* = 9.7150 (8) Å
*b* = 11.3607 (9) Å
*c* = 16.8591 (14) Å
α = 102.605 (1)°
β = 102.984 (1)°
γ = 104.752 (1)°
*V* = 1676.8 (2) Å^3^
*Z* = 2
Mo *K*α radiation
μ = 0.57 mm^−1^
*T* = 291 K
0.28 × 0.22 × 0.20 mm

#### Data collection

Bruker SMART APEX CCD diffractometer
Absorption correction: multi-scan (*SADABS*; Bruker, 2000 ▶) *T*_min_ = 0.858, *T*_max_ = 0.895
17629 measured reflections
6547 independent reflections
5722 reflections with *I* > \2(*I*)
*R*_int_ = 0.043

#### Refinement

*R*[*F* ^2^ > 2σ(*F* ^2^)] = 0.040
*wR*(*F* ^2^) = 0.111
*S* = 1.01
6547 reflections
445 parameters
H-atom parameters constrained
Δρ_max_ = 0.59 e Å^−3^
Δρ_min_ = −0.23 e Å^−3^

### 

Data collection: *SMART* (Bruker, 2000 ▶); cell refinement: *SAINT-Plus* (Bruker, 2000 ▶); data reduction: *SAINT-Plus*; program(s) used to solve structure: *SHELXTL* (Sheldrick, 2008 ▶); program(s) used to refine structure: *SHELXTL*; molecular graphics: *SHELXTL*; software used to prepare material for publication: *SHELXTL*.

## Supplementary Material

Crystal structure: contains datablock(s) global, I. DOI: 10.1107/S1600536811044588/br2174sup1.cif

Structure factors: contains datablock(s) I. DOI: 10.1107/S1600536811044588/br2174Isup2.hkl

Additional supplementary materials:  crystallographic information; 3D view; checkCIF report

## Figures and Tables

**Table 1:** Hydrogen-bond geometry (Å, °)

*D*—H⋯*A*	*D*—H	H⋯*A*	*D*⋯*A*	*D*—H⋯*A*
C25—H25⋯O5^i^	0.93	2.54	3.386 (3)	151
C29—H29⋯O4^ii^	0.93	2.59	3.450 (3)	153
C34—H34⋯O6^iii^	0.93	2.52	3.363 (3)	151

Symmetry codes: (i) 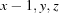; (ii) 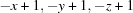; (iii) 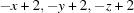.

**Fig. 1.**
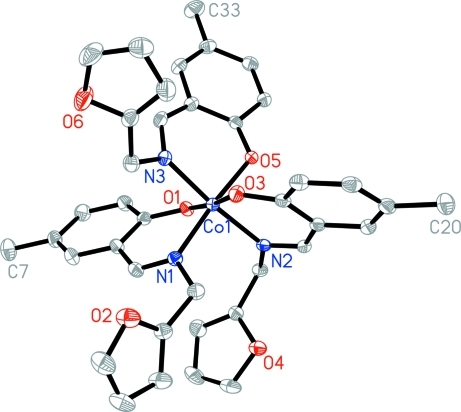
The structure of the title compound (I), with the atom numbering scheme of the unique atoms (30% probability ellipsoids).

**Fig. 2.**
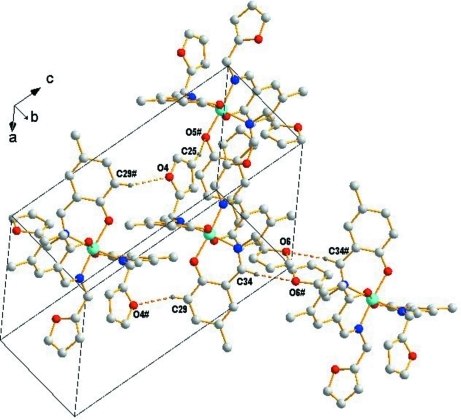
Partial packing view showing the chain formed through C–H···O hydrogen bonds.

## supplementary crystallographic information

### Comment

The Schiff base ligands may act as a bidentate N,O- (Castillo *et al.*, 2003) and a tridentate N,O,O-donor ligand (Erxleben *et al.*, 2001) in the coordination chemistry. In general, the Schiff base metal complexes possess antitumour activities (Ren *et al.*, 2002; Liu *et al.*, 1992) and antimicrobial (Panneerselvam *et al.*, 2005). In addition, cobalt is an important life-required element. For example, vitamin B12, also called cobalamin, which is a water soluble vitamin with a key role in the normal functioning of the brain and nervous system, and for the formation of blood (Randaccio *et al.*, 2010). By taking the biological importance of element cobalt into account, we designed the title complex with the bidentate N,O-donor Schiff base ligands (Scheme I).

The title complex reported here is the mononuclear cobalt(III) complex of the Schiff-base ligand, derived from the condensation of 5-methylsalicylaldehyde and furfuryl amine (Fig. 1). The cobalt(III) atom has a distorted octahedral coordination sphere. Cobalt(III) atom is six-coordinated by three imino N atoms and three phenolic O atoms from three bidentate Schiff-base ligands. Analogous octahedral Co(III) species were previously reported in the literatures (Ray *et al.*, 2008; Sari *et al.*, 1997; Olejnik *et al.*, 1994). All bond lengths are within normal ranges (Allen *et al.*, 1987). It is interesting to point out that the planes of the six-membered chelate rings coordinated to the same Co(III) ion were twisted by 76.41 (3)°, 70.99 (4)°, 84.60 (3)° with respect to each other.

In the crystal structure, the molecules are linked *via* intermolecular C—H···O hydrogen bonds (Fig.2).

### Experimental

5-methylsalicylaldehyde (272 mg, 2 mmol) and furfurylamine (194 mg, 2 mmol) were dissolved in an aqueous methanol solution (25 mL).The mixture was stirred at room temperature for 1 h to give a clear yellow solution, which was added to a solutionof Co(NO_3_)_2_.6H_2_O (291 mg, 1 mmol) in methanol (10 mL). The mixture was stirred for 30 min at room temperature to give a brown solution and then filtered. The red single crystals suitable for X-ray analysis were obtained by slowly evaporating the above filtrate at room temperature. The crystals were isolated and dried in a vacuum desiccator containing anhydrous CaCl_2_, in about 66% yield. Anal. Calcd for C_39_H_36_CoN_3_O_6_: C, 66.76; H, 5.17; N, 5.99. Found: C, 66.52; H, 5.10; N, 5.67%. IR (KBr, cm^-1^): 3445, 2918, 1625, 1535, 1467, 1428, 1385, 1318, 1254, 1217, 1143, 1078, 1017, 905, 819, 741, 598, 455.

### Refinement

All the H atoms were placed in geometrically idealized positions and constrained to ride on their parent atoms, with C—H distances of 0.93–0.97 Å, and with *U*_iso_(H) = 1.2*U*_eq_(carrier) or 1.5*U*_eq_(methyl groups).

### Crystal data

**Table:**

[Co(C_13_H_12_NO_2_)_3_]	*Z* = 2
*M**_r_* = 701.64	*F*(000) = 732
Triclinic, *P*1	*D*_x_ = 1.390 Mg m^−^^3^
Hall symbol: -P 1	Mo *K*α radiation, λ = 0.71073 Å
*a* = 9.7150 (8) Å	Cell parameters from 8681 reflections
*b* = 11.3607 (9) Å	θ = 2.3–28.2°
*c* = 16.8591 (14) Å	µ = 0.57 mm^−^^1^
α = 102.605 (1)°	*T* = 291 K
β = 102.984 (1)°	Block, red
γ = 104.752 (1)°	0.28 × 0.22 × 0.20 mm
*V* = 1676.8 (2) Å^3^	

### Data collection

**Table:**

Bruker SMART APEX CCD diffractometer	6547 independent reflections
Radiation source: fine-focus sealed tube	5722 reflections with *I* > \2(*I*)
graphite	*R*_int_ = 0.043
phi and ω scans	θ_max_ = 26.0°, θ_min_ = 1.9°
Absorption correction: multi-scan (*SADABS*; Bruker, 2000)	*h* = −11→11
*T*_min_ = 0.858, *T*_max_ = 0.895	*k* = −14→14
17629 measured reflections	*l* = −20→20

### Refinement

**Table:**

Refinement on *F*^2^	Primary atom site location: structure-invariant direct methods
Least-squares matrix: full	Secondary atom site location: difference Fourier map
*R*[*F*^2^ > 2σ(*F*^2^)] = 0.040	Hydrogen site location: inferred from neighbouring sites
*wR*(*F*^2^) = 0.111	H-atom parameters constrained
*S* = 1.01	*w* = 1/[σ^2^(*F*_o_^2^) + (0.0698*P*)^2^ + 0.2448*P*] where *P* = (*F*_o_^2^ + 2*F*_c_^2^)/3
6547 reflections	(Δ/σ)_max_ < 0.001
445 parameters	Δρ_max_ = 0.59 e Å^−^^3^
0 restraints	Δρ_min_ = −0.23 e Å^−^^3^

### Special details

**Table:**

Geometry. All esds (except the esd in the dihedral angle between two l.s. planes) are estimated using the full covariance matrix. The cell esds are taken into account individually in the estimation of esds in distances, angles and torsion angles; correlations between esds in cell parameters are only used when they are defined by crystal symmetry. An approximate (isotropic) treatment of cell esds is used for estimating esds involving l.s. planes.
Refinement. Refinement of F^2^ against ALL reflections. The weighted R-factor wR and goodness of fit S are based on F^2^, conventional R-factors R are based on F, with F set to zero for negative F^2^. The threshold expression of F^2^ > 2sigma(F^2^) is used only for calculating R-factors(gt) etc. and is not relevant to the choice of reflections for refinement. R-factors based on F^2^ are statistically about twice as large as those based on F, and R- factors based on ALL data will be even larger.

### Fractional atomic coordinates and isotropic or equivalent isotropic displacement parameters (Å^2^)

**Table:**

	*x*	*y*	*z*	*U*_iso_*/*U*_eq_	
C1	0.4525 (2)	0.84481 (19)	0.81135 (13)	0.0386 (4)	
C2	0.5118 (2)	0.87505 (18)	0.74636 (13)	0.0353 (4)	
C3	0.5290 (3)	0.9974 (2)	0.73886 (15)	0.0451 (5)	
H3	0.5661	1.0197	0.6961	0.054*	
C4	0.4928 (3)	1.0862 (2)	0.79267 (16)	0.0514 (6)	
H4	0.5077	1.1671	0.7860	0.062*	
C5	0.4338 (3)	1.0576 (2)	0.85741 (15)	0.0497 (5)	
C6	0.4135 (3)	0.9373 (2)	0.86463 (15)	0.0486 (5)	
H6	0.3724	0.9154	0.9062	0.058*	
C7	0.3922 (4)	1.1564 (3)	0.91538 (19)	0.0727 (8)	
H7A	0.3105	1.1146	0.9331	0.109*	
H7B	0.3634	1.2131	0.8852	0.109*	
H7C	0.4763	1.2041	0.9645	0.109*	
C8	0.4168 (2)	0.7179 (2)	0.81855 (13)	0.0396 (5)	
H8	0.3528	0.6970	0.8505	0.047*	
C9	0.4039 (3)	0.4991 (2)	0.78844 (14)	0.0443 (5)	
H9A	0.3432	0.4466	0.7315	0.053*	
H9B	0.4866	0.4673	0.8043	0.053*	
C10	0.3131 (2)	0.4805 (2)	0.84705 (14)	0.0421 (5)	
C11	0.1680 (3)	0.4320 (3)	0.83652 (17)	0.0624 (7)	
H11	0.0921	0.4047	0.7853	0.075*	
C12	0.1498 (3)	0.4296 (3)	0.91689 (19)	0.0685 (8)	
H12	0.0608	0.4003	0.9291	0.082*	
C13	0.2842 (3)	0.4771 (3)	0.97078 (18)	0.0705 (8)	
H13	0.3054	0.4871	1.0289	0.085*	
C14	0.5834 (2)	0.37043 (18)	0.62530 (13)	0.0352 (4)	
C15	0.6782 (2)	0.42767 (18)	0.70947 (13)	0.0352 (4)	
C16	0.7650 (2)	0.3586 (2)	0.74421 (15)	0.0437 (5)	
H16	0.8211	0.3898	0.8013	0.052*	
C17	0.7683 (2)	0.2458 (2)	0.69516 (16)	0.0472 (5)	
H17	0.8287	0.2037	0.7197	0.057*	
C18	0.6839 (2)	0.1928 (2)	0.60989 (16)	0.0468 (5)	
C19	0.5893 (2)	0.25399 (19)	0.57714 (15)	0.0424 (5)	
H19	0.5272	0.2176	0.5215	0.051*	
C20	0.7008 (3)	0.0749 (2)	0.5557 (2)	0.0679 (7)	
H20A	0.6445	0.0580	0.4975	0.102*	
H20B	0.6645	0.0036	0.5757	0.102*	
H20C	0.8041	0.0886	0.5597	0.102*	
C21	0.4791 (2)	0.42576 (18)	0.58591 (12)	0.0344 (4)	
H21	0.4158	0.3784	0.5318	0.041*	
C22	0.3441 (2)	0.56849 (19)	0.56579 (13)	0.0364 (4)	
H22A	0.3832	0.6562	0.5662	0.044*	
H22B	0.3141	0.5154	0.5074	0.044*	
C23	0.2122 (2)	0.55384 (19)	0.59660 (12)	0.0369 (4)	
C24	0.1454 (3)	0.6340 (2)	0.62894 (16)	0.0539 (6)	
H24	0.1783	0.7222	0.6416	0.065*	
C25	0.0136 (3)	0.5577 (3)	0.64020 (18)	0.0636 (7)	
H25	−0.0556	0.5867	0.6619	0.076*	
C26	0.0092 (3)	0.4395 (3)	0.61410 (18)	0.0617 (7)	
H26	−0.0660	0.3699	0.6140	0.074*	
C27	0.8930 (2)	0.89061 (19)	0.73095 (13)	0.0390 (4)	
C28	0.8274 (2)	0.78873 (18)	0.65436 (13)	0.0346 (4)	
C29	0.8810 (2)	0.8046 (2)	0.58505 (14)	0.0414 (5)	
H29	0.8437	0.7387	0.5344	0.050*	
C30	0.9870 (2)	0.9150 (2)	0.59038 (14)	0.0449 (5)	
H30	1.0188	0.9215	0.5430	0.054*	
C31	1.0489 (2)	1.0181 (2)	0.66481 (15)	0.0464 (5)	
C32	1.0010 (2)	1.0024 (2)	0.73354 (14)	0.0467 (5)	
H32	1.0416	1.0684	0.7842	0.056*	
C33	1.1623 (3)	1.1385 (2)	0.66737 (19)	0.0683 (7)	
H33A	1.1139	1.1837	0.6345	0.102*	
H33B	1.2374	1.1175	0.6440	0.102*	
H33C	1.2078	1.1911	0.7252	0.102*	
C34	0.8682 (2)	0.8740 (2)	0.80870 (13)	0.0419 (5)	
H34	0.9323	0.9347	0.8587	0.050*	
C35	0.7820 (3)	0.7725 (2)	0.90397 (13)	0.0479 (5)	
H35A	0.7107	0.6934	0.9014	0.057*	
H35B	0.7586	0.8419	0.9370	0.057*	
C36	0.9345 (3)	0.7756 (2)	0.94819 (13)	0.0458 (5)	
C37	1.0248 (3)	0.7123 (3)	0.92707 (18)	0.0725 (8)	
H37	1.0048	0.6495	0.8764	0.087*	
C38	1.1573 (3)	0.7585 (3)	0.9962 (2)	0.0715 (8)	
H38	1.2410	0.7320	0.9997	0.086*	
C39	1.1391 (3)	0.8452 (3)	1.0536 (2)	0.0832 (10)	
H39	1.2100	0.8920	1.1056	0.100*	
Co1	0.60770 (3)	0.65937 (2)	0.719934 (15)	0.03035 (10)	
N1	0.46557 (18)	0.63065 (15)	0.78462 (10)	0.0339 (4)	
N2	0.46406 (16)	0.53381 (15)	0.61766 (10)	0.0308 (3)	
N3	0.76538 (18)	0.78309 (16)	0.81676 (10)	0.0368 (4)	
O1	0.54573 (15)	0.79318 (12)	0.69174 (8)	0.0353 (3)	
O2	0.38919 (19)	0.51014 (19)	0.93061 (10)	0.0629 (5)	
O3	0.68839 (15)	0.53894 (13)	0.75724 (9)	0.0392 (3)	
O4	0.13047 (17)	0.43143 (15)	0.58673 (10)	0.0505 (4)	
O5	0.73163 (14)	0.68008 (12)	0.64728 (9)	0.0366 (3)	
O6	1.0013 (2)	0.85831 (19)	1.02703 (12)	0.0764 (6)	

### Atomic displacement parameters (Å^2^)

**Table:**

	*U*^11^	*U*^22^	*U*^33^	*U*^12^	*U*^13^	*U*^23^
C1	0.0399 (11)	0.0405 (11)	0.0402 (11)	0.0153 (9)	0.0176 (9)	0.0125 (9)
C2	0.0309 (10)	0.0351 (10)	0.0389 (10)	0.0106 (8)	0.0097 (8)	0.0095 (8)
C3	0.0508 (13)	0.0388 (11)	0.0531 (13)	0.0158 (10)	0.0233 (11)	0.0182 (10)
C4	0.0569 (14)	0.0367 (11)	0.0645 (15)	0.0193 (10)	0.0203 (12)	0.0152 (11)
C5	0.0506 (13)	0.0475 (13)	0.0524 (13)	0.0227 (11)	0.0175 (11)	0.0066 (10)
C6	0.0536 (13)	0.0542 (13)	0.0469 (13)	0.0233 (11)	0.0259 (11)	0.0137 (10)
C7	0.091 (2)	0.0616 (17)	0.0735 (19)	0.0401 (16)	0.0331 (16)	0.0066 (14)
C8	0.0405 (11)	0.0462 (12)	0.0378 (11)	0.0142 (9)	0.0191 (9)	0.0155 (9)
C9	0.0501 (13)	0.0374 (11)	0.0477 (12)	0.0097 (9)	0.0199 (10)	0.0166 (9)
C10	0.0450 (12)	0.0420 (11)	0.0426 (11)	0.0109 (9)	0.0143 (9)	0.0208 (9)
C11	0.0426 (13)	0.0807 (18)	0.0606 (16)	0.0075 (13)	0.0137 (12)	0.0301 (14)
C12	0.0568 (16)	0.089 (2)	0.083 (2)	0.0262 (15)	0.0389 (15)	0.0490 (17)
C13	0.079 (2)	0.100 (2)	0.0548 (16)	0.0344 (18)	0.0339 (15)	0.0466 (16)
C14	0.0303 (10)	0.0326 (10)	0.0433 (11)	0.0076 (8)	0.0144 (8)	0.0113 (8)
C15	0.0279 (9)	0.0347 (10)	0.0461 (11)	0.0094 (8)	0.0150 (8)	0.0146 (9)
C16	0.0354 (11)	0.0427 (12)	0.0554 (13)	0.0121 (9)	0.0121 (9)	0.0208 (10)
C17	0.0352 (11)	0.0394 (11)	0.0759 (16)	0.0161 (9)	0.0201 (11)	0.0256 (11)
C18	0.0409 (12)	0.0324 (11)	0.0723 (16)	0.0111 (9)	0.0271 (11)	0.0154 (10)
C19	0.0385 (11)	0.0344 (10)	0.0516 (13)	0.0072 (9)	0.0171 (10)	0.0084 (9)
C20	0.0647 (17)	0.0470 (14)	0.095 (2)	0.0260 (13)	0.0314 (16)	0.0095 (14)
C21	0.0289 (9)	0.0348 (10)	0.0344 (10)	0.0044 (8)	0.0101 (8)	0.0063 (8)
C22	0.0311 (10)	0.0417 (11)	0.0372 (10)	0.0108 (8)	0.0086 (8)	0.0152 (9)
C23	0.0331 (10)	0.0424 (11)	0.0348 (10)	0.0135 (9)	0.0065 (8)	0.0128 (8)
C24	0.0497 (13)	0.0567 (14)	0.0613 (15)	0.0284 (11)	0.0151 (11)	0.0170 (12)
C25	0.0459 (14)	0.096 (2)	0.0695 (17)	0.0415 (15)	0.0273 (12)	0.0310 (15)
C26	0.0373 (13)	0.0832 (19)	0.0700 (17)	0.0126 (12)	0.0246 (12)	0.0320 (15)
C27	0.0364 (11)	0.0373 (11)	0.0389 (11)	0.0080 (9)	0.0096 (9)	0.0086 (9)
C28	0.0310 (10)	0.0352 (10)	0.0395 (11)	0.0118 (8)	0.0131 (8)	0.0104 (8)
C29	0.0403 (11)	0.0402 (11)	0.0397 (11)	0.0079 (9)	0.0149 (9)	0.0065 (9)
C30	0.0424 (12)	0.0491 (12)	0.0476 (12)	0.0124 (10)	0.0201 (10)	0.0188 (10)
C31	0.0407 (12)	0.0401 (11)	0.0554 (13)	0.0068 (9)	0.0114 (10)	0.0178 (10)
C32	0.0460 (12)	0.0356 (11)	0.0456 (12)	0.0030 (9)	0.0059 (10)	0.0059 (9)
C33	0.0650 (17)	0.0520 (15)	0.0751 (18)	−0.0044 (13)	0.0181 (14)	0.0232 (13)
C34	0.0422 (11)	0.0373 (11)	0.0350 (10)	0.0061 (9)	0.0061 (9)	0.0018 (8)
C35	0.0476 (13)	0.0591 (14)	0.0341 (11)	0.0140 (11)	0.0126 (9)	0.0110 (10)
C36	0.0479 (12)	0.0470 (12)	0.0360 (11)	0.0097 (10)	0.0081 (9)	0.0102 (9)
C37	0.0686 (18)	0.084 (2)	0.0575 (16)	0.0322 (16)	0.0133 (14)	0.0033 (14)
C38	0.0537 (16)	0.081 (2)	0.082 (2)	0.0272 (15)	0.0139 (15)	0.0284 (17)
C39	0.0629 (18)	0.083 (2)	0.072 (2)	0.0241 (16)	−0.0195 (15)	−0.0003 (16)
Co1	0.02797 (15)	0.03043 (15)	0.03159 (16)	0.00821 (11)	0.00945 (11)	0.00764 (11)
N1	0.0350 (9)	0.0347 (8)	0.0327 (8)	0.0087 (7)	0.0111 (7)	0.0127 (7)
N2	0.0246 (8)	0.0355 (8)	0.0315 (8)	0.0069 (6)	0.0092 (6)	0.0108 (7)
N3	0.0369 (9)	0.0391 (9)	0.0301 (8)	0.0099 (7)	0.0088 (7)	0.0055 (7)
O1	0.0390 (7)	0.0360 (7)	0.0372 (7)	0.0150 (6)	0.0174 (6)	0.0135 (6)
O2	0.0504 (10)	0.0905 (13)	0.0486 (10)	0.0127 (9)	0.0126 (8)	0.0361 (9)
O3	0.0381 (8)	0.0374 (7)	0.0388 (8)	0.0152 (6)	0.0048 (6)	0.0080 (6)
O4	0.0419 (8)	0.0481 (9)	0.0615 (10)	0.0090 (7)	0.0222 (7)	0.0150 (7)
O5	0.0318 (7)	0.0334 (7)	0.0395 (7)	0.0052 (6)	0.0155 (6)	0.0014 (6)
O6	0.0728 (12)	0.0761 (13)	0.0538 (11)	0.0312 (10)	−0.0110 (9)	−0.0110 (9)

### Geometric parameters (Å, °)

**Table:**

C1—C2	1.413 (3)	C22—N2	1.485 (2)
C1—C6	1.414 (3)	C22—H22A	0.9700
C1—C8	1.433 (3)	C22—H22B	0.9700
C2—O1	1.319 (2)	C23—C24	1.338 (3)
C2—C3	1.395 (3)	C23—O4	1.367 (2)
C3—C4	1.374 (3)	C24—C25	1.429 (4)
C3—H3	0.9300	C24—H24	0.9300
C4—C5	1.402 (3)	C25—C26	1.305 (4)
C4—H4	0.9300	C25—H25	0.9300
C5—C6	1.366 (3)	C26—O4	1.375 (3)
C5—C7	1.520 (3)	C26—H26	0.9300
C6—H6	0.9300	C27—C32	1.412 (3)
C7—H7A	0.9600	C27—C28	1.420 (3)
C7—H7B	0.9600	C27—C34	1.428 (3)
C7—H7C	0.9600	C28—O5	1.305 (2)
C8—N1	1.284 (3)	C28—C29	1.409 (3)
C8—H8	0.9300	C29—C30	1.376 (3)
C9—C10	1.479 (3)	C29—H29	0.9300
C9—N1	1.483 (2)	C30—C31	1.403 (3)
C9—H9A	0.9700	C30—H30	0.9300
C9—H9B	0.9700	C31—C32	1.371 (3)
C10—C11	1.331 (3)	C31—C33	1.507 (3)
C10—O2	1.357 (3)	C32—H32	0.9300
C11—C12	1.410 (4)	C33—H33A	0.9600
C11—H11	0.9300	C33—H33B	0.9600
C12—C13	1.309 (4)	C33—H33C	0.9600
C12—H12	0.9300	C34—N3	1.296 (3)
C13—O2	1.366 (3)	C34—H34	0.9300
C13—H13	0.9300	C35—N3	1.477 (3)
C14—C15	1.411 (3)	C35—C36	1.489 (3)
C14—C19	1.415 (3)	C35—H35A	0.9700
C14—C21	1.438 (3)	C35—H35B	0.9700
C15—O3	1.309 (2)	C36—C37	1.329 (4)
C15—C16	1.409 (3)	C36—O6	1.352 (3)
C16—C17	1.377 (3)	C37—C38	1.416 (4)
C16—H16	0.9300	C37—H37	0.9300
C17—C18	1.394 (3)	C38—C39	1.299 (4)
C17—H17	0.9300	C38—H38	0.9300
C18—C19	1.377 (3)	C39—O6	1.370 (4)
C18—C20	1.516 (3)	C39—H39	0.9300
C19—H19	0.9300	Co1—O1	1.8848 (13)
C20—H20A	0.9600	Co1—O3	1.8927 (13)
C20—H20B	0.9600	Co1—O5	1.9104 (13)
C20—H20C	0.9600	Co1—N3	1.9405 (16)
C21—N2	1.286 (2)	Co1—N2	1.9459 (16)
C21—H21	0.9300	Co1—N1	1.9505 (16)
C22—C23	1.472 (3)		
			
C2—C1—C6	119.64 (19)	C25—C24—H24	126.6
C2—C1—C8	120.94 (18)	C26—C25—C24	106.6 (2)
C6—C1—C8	119.10 (19)	C26—C25—H25	126.7
O1—C2—C3	119.33 (18)	C24—C25—H25	126.7
O1—C2—C1	123.68 (17)	C25—C26—O4	111.1 (2)
C3—C2—C1	116.94 (18)	C25—C26—H26	124.4
C4—C3—C2	122.1 (2)	O4—C26—H26	124.4
C4—C3—H3	118.9	C32—C27—C28	119.94 (19)
C2—C3—H3	118.9	C32—C27—C34	118.93 (19)
C3—C4—C5	121.6 (2)	C28—C27—C34	120.43 (18)
C3—C4—H4	119.2	O5—C28—C29	119.33 (17)
C5—C4—H4	119.2	O5—C28—C27	124.00 (18)
C6—C5—C4	117.1 (2)	C29—C28—C27	116.42 (18)
C6—C5—C7	122.1 (2)	C30—C29—C28	121.7 (2)
C4—C5—C7	120.8 (2)	C30—C29—H29	119.1
C5—C6—C1	122.6 (2)	C28—C29—H29	119.1
C5—C6—H6	118.7	C29—C30—C31	122.3 (2)
C1—C6—H6	118.7	C29—C30—H30	118.8
C5—C7—H7A	109.5	C31—C30—H30	118.8
C5—C7—H7B	109.5	C32—C31—C30	116.5 (2)
H7A—C7—H7B	109.5	C32—C31—C33	122.8 (2)
C5—C7—H7C	109.5	C30—C31—C33	120.7 (2)
H7A—C7—H7C	109.5	C31—C32—C27	123.0 (2)
H7B—C7—H7C	109.5	C31—C32—H32	118.5
N1—C8—C1	125.56 (18)	C27—C32—H32	118.5
N1—C8—H8	117.2	C31—C33—H33A	109.5
C1—C8—H8	117.2	C31—C33—H33B	109.5
C10—C9—N1	117.47 (18)	H33A—C33—H33B	109.5
C10—C9—H9A	107.9	C31—C33—H33C	109.5
N1—C9—H9A	107.9	H33A—C33—H33C	109.5
C10—C9—H9B	107.9	H33B—C33—H33C	109.5
N1—C9—H9B	107.9	N3—C34—C27	126.66 (19)
H9A—C9—H9B	107.2	N3—C34—H34	116.7
C11—C10—O2	109.2 (2)	C27—C34—H34	116.7
C11—C10—C9	134.3 (2)	N3—C35—C36	113.19 (18)
O2—C10—C9	116.36 (19)	N3—C35—H35A	108.9
C10—C11—C12	107.7 (2)	C36—C35—H35A	108.9
C10—C11—H11	126.1	N3—C35—H35B	108.9
C12—C11—H11	126.1	C36—C35—H35B	108.9
C13—C12—C11	105.9 (2)	H35A—C35—H35B	107.8
C13—C12—H12	127.1	C37—C36—O6	109.5 (2)
C11—C12—H12	127.1	C37—C36—C35	133.6 (2)
C12—C13—O2	111.3 (2)	O6—C36—C35	116.9 (2)
C12—C13—H13	124.4	C36—C37—C38	107.1 (3)
O2—C13—H13	124.4	C36—C37—H37	126.4
C15—C14—C19	119.53 (19)	C38—C37—H37	126.4
C15—C14—C21	122.40 (18)	C39—C38—C37	106.5 (3)
C19—C14—C21	118.06 (19)	C39—C38—H38	126.7
O3—C15—C16	118.42 (19)	C37—C38—H38	126.7
O3—C15—C14	124.25 (18)	C38—C39—O6	110.8 (3)
C16—C15—C14	117.32 (19)	C38—C39—H39	124.6
C17—C16—C15	121.1 (2)	O6—C39—H39	124.6
C17—C16—H16	119.4	O1—Co1—O3	173.72 (6)
C15—C16—H16	119.4	O1—Co1—O5	87.15 (6)
C16—C17—C18	122.1 (2)	O3—Co1—O5	92.04 (6)
C16—C17—H17	119.0	O1—Co1—N3	89.22 (7)
C18—C17—H17	119.0	O3—Co1—N3	84.56 (7)
C19—C18—C17	117.3 (2)	O5—Co1—N3	91.16 (6)
C19—C18—C20	122.1 (2)	O1—Co1—N2	92.27 (6)
C17—C18—C20	120.6 (2)	O3—Co1—N2	93.83 (6)
C18—C19—C14	122.3 (2)	O5—Co1—N2	83.72 (6)
C18—C19—H19	118.9	N3—Co1—N2	174.59 (6)
C14—C19—H19	118.9	O1—Co1—N1	91.47 (6)
C18—C20—H20A	109.5	O3—Co1—N1	89.89 (6)
C18—C20—H20B	109.5	O5—Co1—N1	174.71 (6)
H20A—C20—H20B	109.5	N3—Co1—N1	93.93 (7)
C18—C20—H20C	109.5	N2—Co1—N1	91.23 (6)
H20A—C20—H20C	109.5	C8—N1—C9	119.34 (17)
H20B—C20—H20C	109.5	C8—N1—Co1	122.97 (14)
N2—C21—C14	126.83 (18)	C9—N1—Co1	117.52 (13)
N2—C21—H21	116.6	C21—N2—C22	116.91 (16)
C14—C21—H21	116.6	C21—N2—Co1	122.58 (13)
C23—C22—N2	113.19 (16)	C22—N2—Co1	119.87 (13)
C23—C22—H22A	108.9	C34—N3—C35	115.85 (18)
N2—C22—H22A	108.9	C34—N3—Co1	122.56 (14)
C23—C22—H22B	108.9	C35—N3—Co1	121.41 (14)
N2—C22—H22B	108.9	C2—O1—Co1	122.20 (12)
H22A—C22—H22B	107.8	C10—O2—C13	105.88 (19)
C24—C23—O4	109.67 (19)	C15—O3—Co1	125.90 (12)
C24—C23—C22	134.5 (2)	C23—O4—C26	105.85 (18)
O4—C23—C22	115.65 (17)	C28—O5—Co1	123.52 (12)
C23—C24—C25	106.7 (2)	C36—O6—C39	106.1 (2)
C23—C24—H24	126.6		

### Hydrogen-bond geometry (Å, °)

**Table:**

*D*—H···*A*	*D*—H	H···*A*	*D*···*A*	*D*—H···*A*
C25—H25···O5^i^	0.93	2.54	3.386 (3)	151
C29—H29···O4^ii^	0.93	2.59	3.450 (3)	153
C34—H34···O6^iii^	0.93	2.52	3.363 (3)	151

Symmetry codes: (i) *x*−1, *y*, *z*; (ii) −*x*+1, −*y*+1, −*z*+1; (iii) −*x*+2, −*y*+2, −*z*+2.

## References

[bb1] Allen, F. H., Kennard, O., Watson, D. G., Brammer, L., Orpen, A. G. & Taylor, R. (1987). *J. Chem. Soc. Perkin Trans. 2*, pp. S1–19.

[bb2] Bruker (2000). *SMART*, *SAINT-Plus* and *SADABS* Bruker AXS Inc., Madison, Wisconsin, USA.

[bb3] Castillo, I., Fernandez-Gonzalez, J. M. & Garate-Morales, J. L. (2003). *J. Mol. Struct.* **657**, 25–35.

[bb4] Erxleben, A. & Schumacher, D. (2001). *Eur. J. Inorg. Chem.* pp. 3039–3046.

[bb5] Liu, M. C., Lin, T. S. & Sartorelli, A. C. (1992). *J. Med. Chem.* **35**, 3672–3677. 10.1021/jm00098a012 1433178

[bb6] Olejnik, Z. & Lis, T. (1994). *Bull. Pol. Acad. Sci. Chem.* **42**, 41–47.

[bb7] Panneerselvam, P., Nair, R. R., Vijayalakshmi, G., Subramanian, E. H. & Sridhar, S. K. (2005). *Eur. J. Med. Chem.* **40**, 225–229. 10.1016/j.ejmech.2004.09.003 15694658

[bb8] Randaccio, L., Geremia, S., Demitri, N. & Wuerges, J. (2010). *Molecules*, **15**, 3228–3259. 10.3390/molecules15053228 PMC6257451 20657474

[bb9] Ray, A., Banerjee, S., Rosair, G. M., Gramlich, V. & Mitra, S. (2008). *Struct. Chem.* **19**, 459–465.

[bb10] Ren, S., Wang, R., Komatsu, K., Bonaz-Krause, P., Zyrianov, Y., McKenna, C. E., Csipke, C., Tokes, Z. A. & Lien, E. J. (2002). *J. Med. Chem.* **45**, 410–419. 10.1021/jm010252q 11784145

[bb11] Sari, M., Ercan, F., Yagbasan, R., Atakol, O. & Kenar, A. (1997). *Z. Kristallogr. New Cryst. Struct.* **212**, 185–186.

[bb12] Sheldrick, G. M. (2008). *Acta Cryst.* A**64**, 112–122. 10.1107/S0108767307043930 18156677

